# Supplementary material for: Dissecting the role of non-coding RNAs in the accumulation of amyloid and tau neuropathologies in Alzheimer’s disease
Source: Mol Neurodegener. 2017 Jul 1;12:51. doi: 10.1186/s13024-017-0191-y (PMC5494142; doi:10.1186/s13024-017-0191-y)
Supplement: Supplementary file 1 — Demographic information. Table S3. A table of the correlations between each of the pertinent covariates, Study, Age, Sex, NNLS, PMI, RIN, NP, NFT and AD. Figure S1. Behavior of miRNAs implicated in other experiments. Figure S2. Differentially expressed miRNAs and lincRNAs. Figure S3. Validation of RIN association. (DOCX 483 kb) [file 13024_2017_191_MOESM1_ESM.docx]

## Table S1 Demographic information

| Study, (ROS, MAP) | (379, 321) |
| --- | --- |
| Age, mean (sd) | 88.17 (6.5) |
| Sex, (female, male) | (447, 253) |
| PMI, mean (sd) | 7.51 (5.9) |
| NNLS, mean (sd) | 0.43 (0.06) |
| RIN, mean (sd) | 6.52 (1.6) |
| NP, mean (sd) | 0.7 (0.52) |
| NFT, mean (sd) | 0.67 (0.42) |
| Pathological AD (not AD, AD) | (273, 427) |

## Table S3 A table of the correlations between each of the pertinent covariates, Study, Age, Sex, NNLS, PMI, RIN, NP, NFT and AD .

|  | Study | Age | Sex | NNLS | PMI | RIN | NP | NFT | AD |
| --- | --- | --- | --- | --- | --- | --- | --- | --- | --- |
| Study | 1 | -0.11 | -0.02 | -0.09 | 0 | 0.04 | 0.03 | -0.06 | 0.03 |
| Age | -0.11 | 1 | -0.21 | 0.14 | -0.02 | 0.01 | 0.14 | 0.26 | 0.23 |
| Sex | -0.02 | -0.21 | 1 | -0.08 | 0.03 | 0.06 | -0.15 | -0.18 | -0.09 |
| NNLS | -0.09 | 0.14 | -0.08 | 1 | 0.07 | -0.39 | 0.11 | 0.11 | 0.1 |
| PMI | 0 | -0.02 | 0.03 | 0.07 | 1 | -0.12 | -0.02 | -0.03 | -0.01 |
| RIN | 0.04 | 0.01 | 0.06 | -0.39 | -0.12 | 1 | -0.16 | -0.15 | -0.12 |
| NP | 0.03 | 0.14 | -0.15 | 0.11 | -0.02 | -0.16 | 1 | 0.59 | 0.81 |
| NFT | -0.06 | 0.26 | -0.18 | 0.11 | -0.03 | -0.15 | 0.59 | 1 | 0.52 |
| AD | 0.03 | 0.23 | -0.09 | 0.1 | -0.01 | -0.12 | 0.81 | 0.52 | 1 |

## Figure S1 Behavior of miRNAs implicated in other experiments

The relationship between average miRNA expression and their association with pathological AD in ROSMAP is investigated in a). The average expression of a miRNA is plotted by the x-axis. The z-score of a test for the association between a miRNA and AD after correcting for other factors is plotted on the y-axis. miRNA implicated in four other experiments b) prefrontal cortex and hippocampus by Lau et al (Lau *et al.*, 2013), c) the medial frontal gyrus and hippocampus by Cogswell et al (Cogswell *et al.*, 2008), d) the temporal Cortex by Hébert et al (Hébert *et al.*, 2008) and e) the parietal lobe cortex by Nunez-Iglesias et al (Nunez-Iglesias *et al.*, 2010) were examined to verify their relationship with pathological AD in our cohort. Red upwards pointing triangles represent miRNA that were reported as positively associated with AD and blue downwards facing triangles represent miRNA that were reported as negatively associated with AD. The dotted horizontal lines mark the z-score that is equivalent to a Bonferroni p-value cut-off of 0.05.


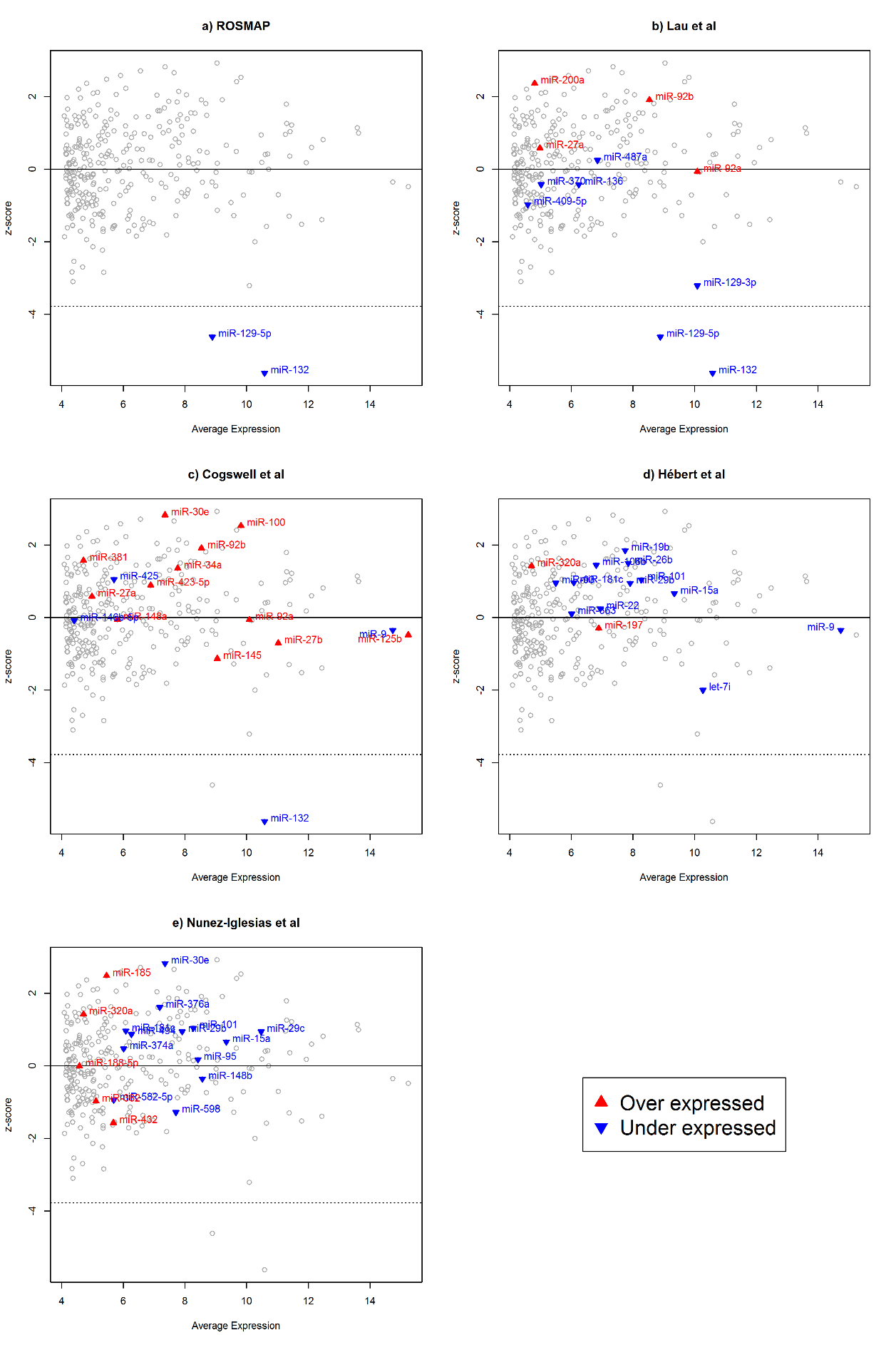


## Figure S2 Differentially expressed miRNAs and lincRNAs

The relationship between a) miRNA or b) lincRNA expression and all the pertinent covariates are explored for the differentially expressed miRNAs and lincRNAs that were listed in Table 1 and Table 4. For each miRNA or lincRNA a linear model is fitted using the study of origin (Study), age at death (Age), sex, neuronal composition (NNLS), post-mortem interval (PMI), RNA integrity number (RIN), neurotic plaques (NP) and neurofibrillary tangles (NFT) as explanatory variables. The standardized effect size of each of these explanatory variables and 95% confidence intervals are then plotted.


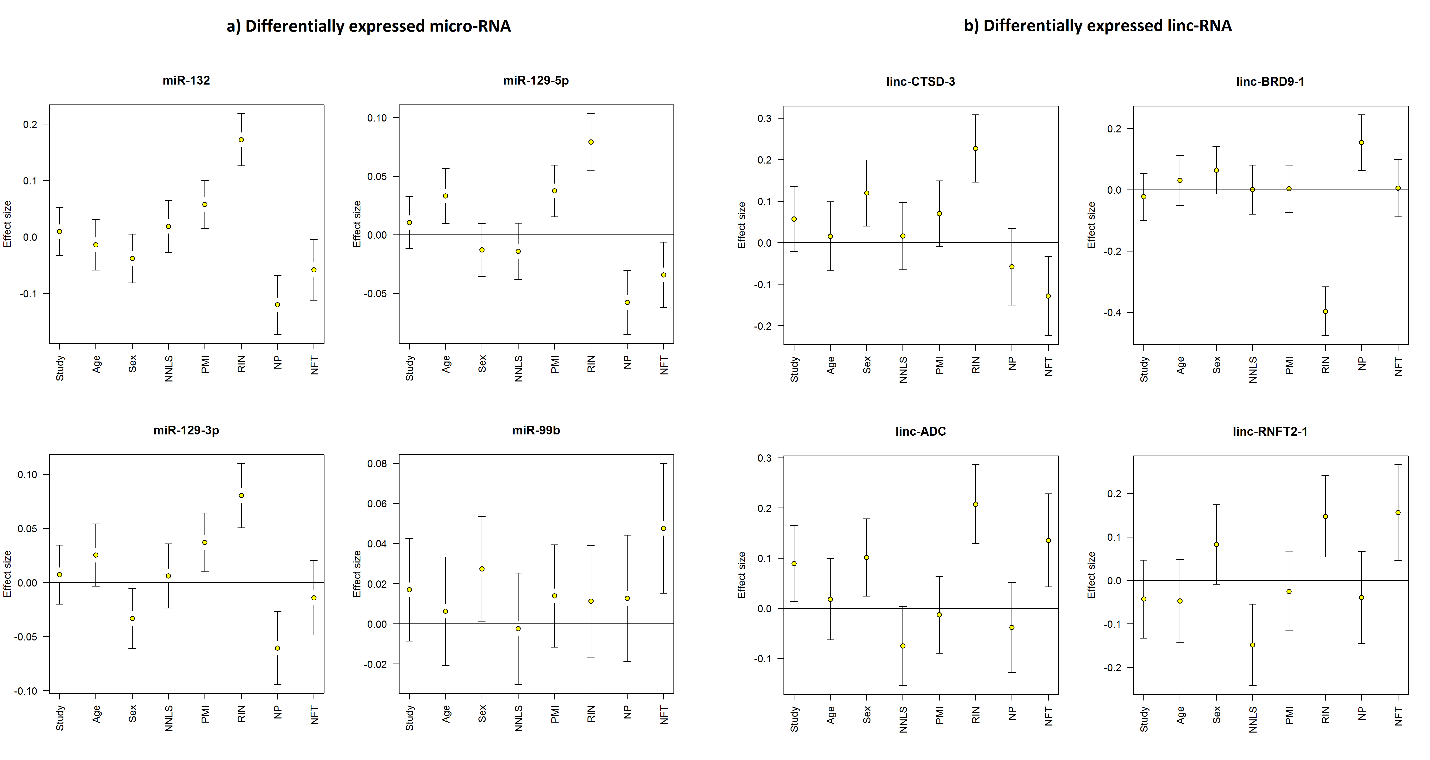


## Figure S3 Validation of RIN association

Many miRNA are observed to have a strong association with RNA-integrity in this cohort, the strongest of which is miR-1260 (Pearson correlation of -0.73). To validate that these associations are not an artifact of the Nanostring platform the expression of miR-1260 was measure via qRT-PCR for 20 subjects. The expression of miR-1260 was normalized to miR-99a, a miRNA that did not show a strong association with RIN. We observed that miR-1260 also has a strong association with RIN (Pearson correlation -0.75) in the qRT-PCR data.

References

Cogswell JP, Ward J, Taylor IA, Waters M, Shi Y, Cannon B*, et al.* Identification of miRNA changes in Alzheimer's disease brain and CSF yields putative biomarkers and insights into disease pathways. Journal of Alzheimer's disease : JAD 2008; 14(1): 27-41.

Hébert SSS, Horré K, Nicolaï L, Papadopoulou AS, Mandemakers W, Silahtaroglu AN*, et al.* Loss of microRNA cluster miR-29a/b-1 in sporadic Alzheimer's disease correlates with increased BACE1/beta-secretase expression. Proceedings of the National Academy of Sciences of the United States of America 2008; 105(17): 6415-20.

Lau P, Bossers K, Janky R, Salta E, Frigerio CS, Barbash S*, et al.* Alteration of the microRNA network during the progression of Alzheimer's disease. EMBO molecular medicine 2013; 5(10): 1613-34.

Nunez-Iglesias J, Liu C-CC, Morgan TE, Finch CE, Zhou XJ. Joint genome-wide profiling of miRNA and mRNA expression in Alzheimer's disease cortex reveals altered miRNA regulation. PloS one 2010; 5(2).
